# Minimizing contrast media dose in CT pulmonary angiography with high-pitch technique

**DOI:** 10.1259/bjr.20190995

**Published:** 2020-05-21

**Authors:** Hanan Alobeidi, Muhammed Alshamari, Jonas Widell, Tomas Eriksson, Mats Lidén

**Affiliations:** 1 Department of Radiology, Örebro university Hospital, Region Örebro län, S-70185 Örebro, Sweden; 2 Department of Radiology, Faculty of Medicine and Health, Örebro University, S-70182 Örebro, Sweden

## Abstract

**Objectives:**

To perform CT pulmonary angiography (CTPA) using a minimal amount of iodinated contrast media.

**Methods:**

47 patients (25 females) with mean age 69 years (range 41–82 years) referred for contrast-enhanced chest CT were prospectively included in this Phase IV clinical drug trial. All participants underwent a study specific CTPA in addition to the chest CT. The participants received 80 mg I/kg body weight Iohexol contrast media using a preparatory saline bolus, a dual flow contrast/saline bolus and a saline flush, and a scanner protocol with 80 kVp dual source high-pitch mode. Three readers independently assessed the image quality on the 3-point scale non-diagnostic, adequate or good-excellent image quality. Additionally, the pulmonary arterial contrast opacification was measured.

**Results:**

On average, the patients received 16.8 ml Iohexol 350 mg I/mL (range 12–20 ml). Mean patient weight was 71 kg (range 50–85 kg). Identically for all readers, pulmonary embolism (PE) was detected in 1/47 participants. The median number of examinations visually scored concerning pulmonary embolism as good–excellent was 47/47 (range 44–47); adequate 0/47 (0–3) and non-diagnostic 0/47 (range 0–0). The proportion adequate or better examinations was for all readers 47/47, 100% [95% confidence interval 92–100%]. The mean attenuation ± standard deviation in the pulmonary trunk was 325 ± 72 Hounsfield unit (range 165–531 Hounsfield unit).

**Conclusions:**

Diagnostic CTPA with 17 ml contrast media is possible in non-obese patients using low kVp, high pitch and carefully designed contrast media administration.

**Advances in knowledge:**

By combining several procedures in a CTPA protocol, the contrast media dose can be minimized.

## Introduction

Pulmonary embolism (PE) is a common condition with high mortality and morbidity.^1,2^ Since the 1990s, CT pulmonary angiography (CTPA) has become the method of choice for imaging in suspected PE.^2–4^ CTPA is a standard procedure that obtains a CT volume while intravenously injected iodinated contrast media (CM) opacifies the pulmonary arteries.

Recent guidelines conclude that the risk of post-contrast acute kidney injury (PC-AKI) is lower than previously believed, but does exist, especially for patients with severely impaired renal function.^5^ For patients at risk for PC-AKI, lowering the CM dose is preferable^6–8^ but the image quality needs to be maintained. The increased attenuation of iodine in low-kVp CT protocols is used to reduce the amount of CM administered in CTPA.^9–17^

Technical developments in CT systems have allowed CTPA with significantly lower CM doses than previously possible. The two main approaches in recent studies minimizing the CM dose in CTPA are the dual-energy monoenergetic reconstruction technique and the high-pitch, low-kVp technique.^14,15,17,18^ Dual-energy techniques rely on advanced image reconstruction from dual-energy data. High-pitch techniques rely on the exact timing of a fast scan during CM transit, where dual-source CT (DSCT) allows a sufficiently high time–current product (mAs) by using both X-ray tubes to produce low-kVp photon beams.

High-pitch CTPA with standard CM dose and bolus triggering has shown excellent image quality.^19^ A significant reduction of CM dose has been achieved with high-pitch protocols even with conventional kVp settings.^20^ A previous study that aimed to minimize CM dose using the high-pitch, low-kVp technique used a test bolus to assess the appropriate delay before the CTPA scan since bolus triggering with a short-contrast bolus time was considered difficult.^14^ However, the test bolus adds to iodine load in the patient and reduces the chance of minimizing the CM. To the best of our knowledge, no previous studies have applied a high-pitch, low-kVp CTPA with bolus triggering and short bolus time.

The purpose of the present study was to develop and validate a bolus-triggered, high-pitch, low-kVp CTPA protocol for ruling out or confirming PE with a minimal amount of CM.

## Methods and materials

### Study design

This Phase IV prospective clinical drug trail (EudraCT 2015-004657-40) was performed after approval from the regional ethics committee (Uppsala,Sweden), the Swedish Medical Products Agency, and the local radiation protection committee. Written informed consent was obtained prior to inclusion.

**Figure 1.**
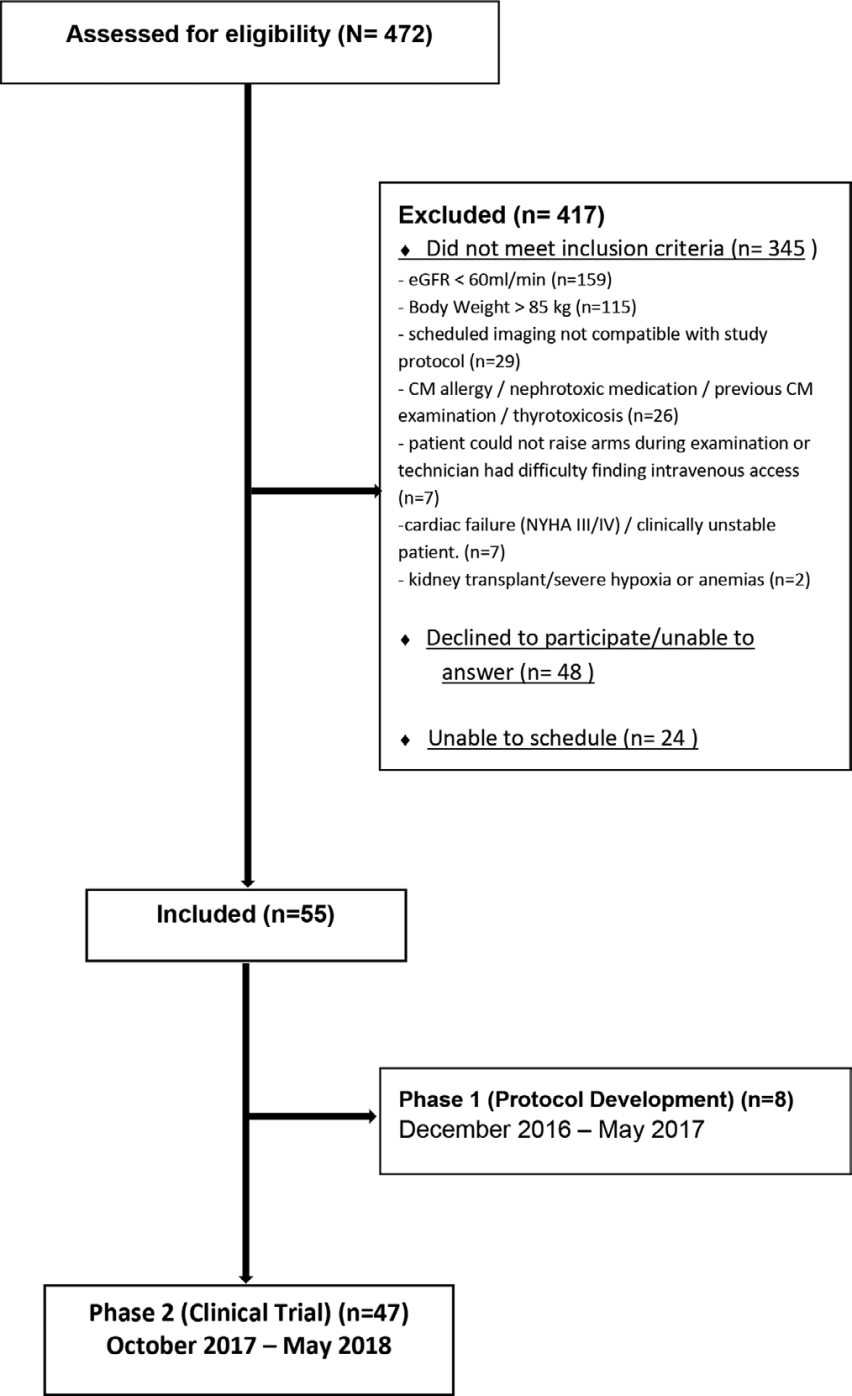
Patient selection flowchart.

From December 2016 to May 2018, 472 patients (males older than 40 and females older than 50 years) referred to the radiology department at Örebro university hospital for contrast-enhanced chest CT were assessed for eligibility. After evaluation of exclusion criteria, according to Figure 1, 55 patients were, during periods of inclusion, consecutively included. The study was conducted in two phases: during the first phase, with eight patients included, the scanner protocol and CM protocol were optimized, after which the protocol parameters were fixed. Thus, 47 participants were included in the second phase, where the diagnostic value of the fixed CTPA protocol was evaluated.

The starting point of the optimization phase was a CM bolus with approximately the same iodine delivery rate (mg iodine/second/kg body weight) as the clinically used 80 kVp CTPA protocol at the study site, but with a significantly shortened bolus time to reduce the CM dose (6 s compared to 13 s). The clinically used 80 kVp protocol at the study site followed previously published results.^10^ Changes during the protocol optimization phase were made based on visual analysis of the distribution of CM in the thoracic vessels in the examination.

The introduction of the preparatory saline bolus from the first patient in the validation cohort was based on an observation during the optimization phase that the CM bolus in some patients seemed to be shortened before it reached the pulmonary arteries. We hypothesized that CM was pooled in collapsed veins, and to avoid pooling, the preparatory bolus was used to distend any collapsed veins, before the injection of the CM bolus.

Baseline characteristics of included subjects are detailed in Table 1. Suspicion of PE was not an inclusion criterion in the trial phase. Consequently, the scheduled examinations that were performed immediately after the study specific CTPA were standard contrast-enhanced chest CT. The main questions for referrals were cancer treatment evaluation or cancer diagnostics. The patients' contribution in the study ended after the CTPA, without study specific follow-up.

**Table 1.** Baseline characteristics of included patients

	Mean ± SD	Range
**Age (years**)	69 ± 8.9	41–82
**Weight (kg**)	71 ± 9.4	50–85
**Height (cm**)	171 ± 8.5	155–189
**BMI (kg/m²**)	24 ± 3.0	17.4–33.3
**e-GFR (ml/min**)	76 ± 11	60–121

SD, standard deviation, BMI, body mass index, e-GFR, estimated glomerular filtration rate.

### Examination technique

The examinations were performed on a second-generation DSCT (Somatom Definition FLASH, Siemens Healthineers) using high-pitch, flash-scan mode. The CTPA scans were acquired in supine position with arms above the head during free breathing. All patients were instructed to breath normally without holding their breath throughout the study examination.

The scan parameters were 80 kVp tube voltage, quality reference tube current of 350 ref-mAs at 80 ref-kV, detector configuration of 128 × 0.6 mm, pitch 1.55, and rotation time 0.28 s. The CT acquisition was triggered by a bolus tracking technique with a 3 s delay (shortest possible) after reaching the threshold of 100 Hounsfield units (HUs) with the region of interest (ROI) placed on the pulmonary trunk. The parameters of the bolus tracking were 80 kVp, 50 mAs, 10 mm slice thickness, and 0.86 s cycle time.

CM was administered through a peripheral venous catheter placed in the antecubital fossa using a Medrad Stellant (Bayer Healthcare) contrast injector. The investigational product was Iohexol 350 mg l ml^−1^ (Omnipaque, GE Healthcare).

The 47 participants in the trial phase received an Iohexol dose corresponding to 80 mg iodine/kg body weight using a triple phase contrast/saline combination as below:

1. A preparatory saline bolus for 8 s, flow rate 0.047 ml/s/(kg body weight).
2. A dual flow of 70% CM/30% NaCl (0.9%) for 7 s, flow rate 0.047 ml/s/(kg body weight), and
3. A saline chaser for 12 s, flow rate 0.047 ml/s/(kg body weight).

For example, a patient with 85 kg body weight received 32 ml saline, followed by 28 ml 70/30 Iohexol 350 mg ml^−1^–saline mix, followed by 48 ml saline, with a 4.0 ml s^−1^ flow rate in all three phases. The 28 ml 70/30 CM/saline mix was composed of 20 ml Iohexol 350 mg I/ml (70%) and 8 ml saline (30%).

The individual CM volumes and injection rates were calculated using a dedicated computer program (OmniVis/OmniJect v. 5.0, distributed in the Nordic countries by GE Healthcare).

### CT image reconstruction

All images were reconstructed with 3 mm/1.5 mm (slice thickness/increment) multiplanar reformat (MPR) on axial, coronal, and sagittal planes, and additional 1 mm/0.7 mm axial images with a soft tissue kernel (I26f5) with strong iterative reconstruction.

## Image quality assessment

### Subjective image quality

All images were independently evaluated by three blinded thoracic and abdominal radiologists [T.E, M.A, and J.W with 37, 11, and 10 years’ experience respectively. The readers did not participate in any part of the planning of the study, inclusion of patients or data analysis. The image quality concerning PE overall and in specific locations in the pulmonary arteries was subjectively scored on a 3-point scale.^11,14^

1. Good to excellent quality, allowing good to excellent diagnosis or exclusion of PE.
2. Adequate quality, inferior quality to those images scored as good or excellent, but still enabling diagnosis or exclusion of PE with relative confidence.
3. Non-diagnostic quality, inferior quality such that the images cannot be used for diagnosis of PE.

### Objective image quality

**Figure 2.**
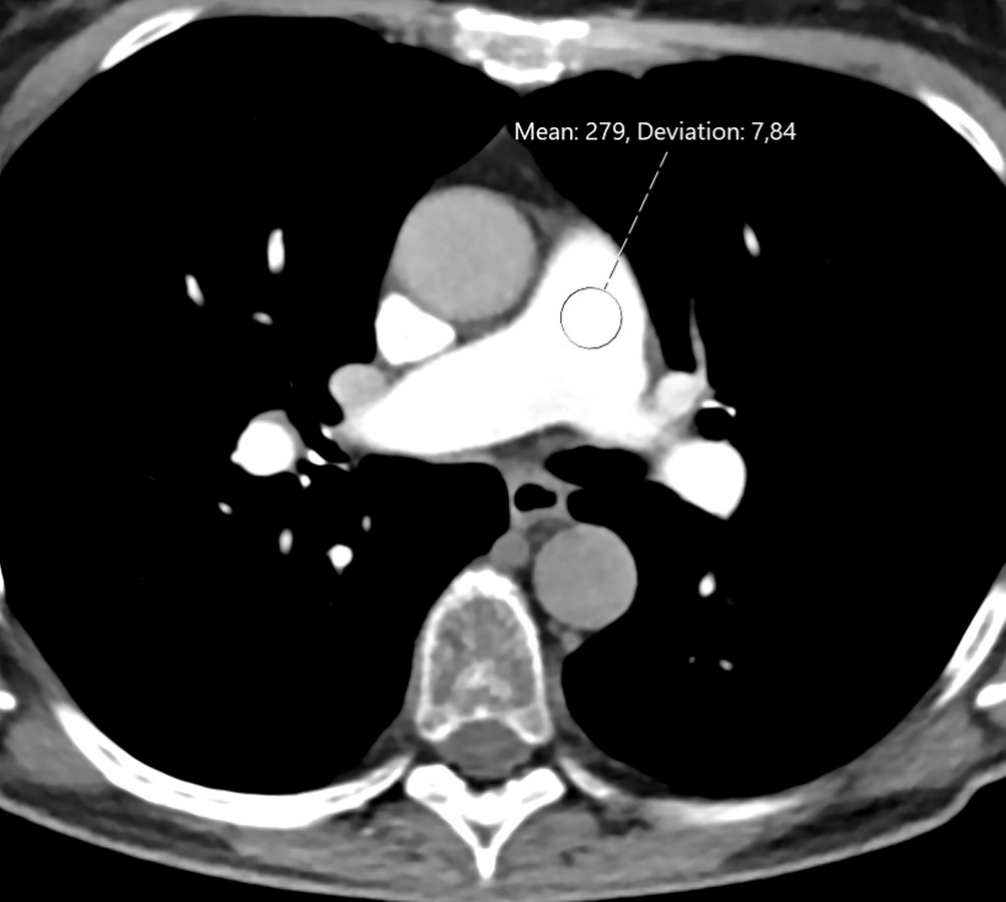
Contrast opacification and image noise in an axial 3 mm slice at the level of the main pulmonary artery in CTPA using 13 ml iohexol 350 mg/ml in a 69-year-old male. The overall image quality was subjectively scored as good–excellent by all readers. CTPA, CT pulmonary angiography.

The contrast opacification in the pulmonary arteries was measured in the 3 mm axial images by placing a ROI in the main pulmonary artery (~1.5 cm diameter), the left and right pulmonary artery (~1 cm), and smaller ROIs in the peripheral arteries including the right upper and lower lobe arteries, left upper and lower lobe arteries, and left lower segmental arteries, see Figure 2. The background noise was measured as the standard deviation in a 1.5 cm ROI in the pulmonary trunk in the 3 mm axial images. The contrast to noise ratio (CNR) was measured in the pulmonary trunk using the formula:

$$CNR=\frac{attenuation_{Pulmonarytrunk}-attenuation_{skeletalmuscle}}{noise_{Pulmonarytrunk}}$$

The attenuation of the background was measured in a ROI in paraspinal skeletal muscle.^19,21^ The CNR was also computed for patients with body weight below and over 80 kg, respectively, to assess the impact of body weight on the noise and attenuation.

### Statistical analysis

Continuous variables are presented as mean ± standard deviation (SD). For the subjective image quality assessment, the proportion of adequate or better examinations were computed with a confidence interval (CI) of 95% using binomial distribution. Two sample *t*-test was used for comparison of CNR according to body weight. For statistics, Matlab R2018b (The MathWorks Inc., Natick, MA, United States) software was used. The sample size of at least 45 patients in the validation cohort was selected to obtain the lower end of a 95% binomially distributed CI for the proportion successful examinations of at least 85%, with an estimated success rate of 95%. Up to 10 patients were pre-planned to be included in the development phase. The total sample size was consequently 55 subjects.

## Results

### Phase 1—protocol optimization

Eight patients were included in the protocol optimization phase. In subjects 1–4, the contrast bolus time was 6 s with no preparatory saline bolus and no dual flow. In Subject 5, a dual flow 50%/50% CM/saline was introduced. In the last three subjects (subjects 6–8) of the optimization phase, the bolus time was increased to 7 s, a 70%/30% CM/saline dual flow was used and the iodine delivery rate was adjusted to obtain a maximum CM dose of 20 ml for a body weight of 85 kg. The preparatory saline bolus was introduced starting with the first patient in the validation cohort.

### Phase 2—validation

In total, 47 patients (25 females, 50–82 years, mean age 67 years and 22 males, 41–81 years, mean age 70 years) were included in the trial phase. CTPA examinations were successfully performed with no adverse events in all 47 subjects. Other patient characteristics are shown in Table 1. Identically for all readers, PE was detected in 1/47 participants. The low PE prevalence is within the expected range of incidental findings since the included patients were referred for standard chest CT and not CTPA.

The mean amount of Iohexol 350 mg I/ml administered was 16.8 ± 2.3 ml, range 12–20 ml.

The median number of examinations with an overall visual score of good to excellent for PE was 47/47 (range 44–47); adequate 0/47 (range 0–3), and non-diagnostic 0/47 (range 0–0), see Table 2. The proportion of examinations with an overall score of adequate or better for all readers was 47/47, 100% (95% CI 92–100%].

**Table 2.** Subjective image quality score concerning PE

Pulmonary arteries	Good–Excellent median [range]	Adequate median [range]	Non-diagnostic median [range]
**Overall**	47 [44–47]	0 [0–3]	0 [0–0]
**Pulmonary trunk**	46 [45–47]	1 [0–2]	0 [0–0]
**Left pulmonary artery**	46 [46–47]	1 [0–1]	0 [0–0]
**Left lower lobar artery ^a^**	46[45–46]	0 [0–1]	0 [0–0]
**Left lower segmental artery ^a^**	46[45–46]	0 [0–1]	0 [0–0]
**Left upper lobar artery**	46 [44–47]	0 [0–2]	1 [0–1]
**Left upper segmental artery**	45[42–46]	1[1–2]	1 [0–3]
**Right pulmonary artery**	46[46–47]	1 [0–1]	0 [0–0]
**Right lower lobar artery ^b^**	46[45–46]	0 [0–1]	0 [0–0]
**Right lower segmental artery ^b^**	46[45–46]	0 [0–1]	0 [0–0]
**Right middle lobar artery**	47[45–47]	0 [0–2]	0 [0–0]
**Right middle segmental artery**	46[44–47]	0 [0–1]	1 [0–2]
**Right upper lobar artery^c^**	45[43–45]	0 [0–2]	0 [0–0]
**Right upper segmental artery ^c^**	45[40–45]	0 [0–4]	0 [0–1]

PE, pulmonary embolism.

a One case underwent a left lower lobectomy, both left lower lobar and lower segmental arteries are missing.

b One case underwent right lower lobectomy, both lower lobar and lower segmental arteries are missing.

c Two cases underwent a right upper lobectomy, both right upper lobar and upper segmental arteries are missing.

The median number of vessel locations scored as non-diagnostic was 3 (range 0–7). The median number of patients with any vessel location scored as non-diagnostic was 2 (range 0–6). The reason for reporting a non-diagnostic score was atelectasis/collapsed segment or small vessel size. No reader reported motion artifacts as reason for non-diagnostic score in any vessel segment.

**Figure 3.**
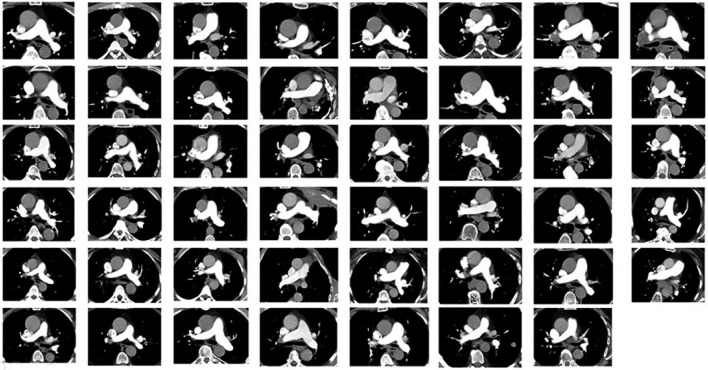
Axial slices at the level of the main pulmonary artery in all 47 patients included in the test cohort (Phase II). The image quality concerning PE was, by all three readers, visually scored as adequate or better in 47/47 patients (100%, 95% confidence interval 92–100%). PE, pulmonary embolism.

The objective image quality assessment showed similar contrast opacification in all evaluated locations of the pulmonary arteries (Table 3). In the pulmonary trunk, the mean ± SD attenuation was 325 ± 72 HU (range 165–531 HU). Figure 3 shows the opacification in the pulmonary trunk in all included subjects. The two examinations with attenuation values <200 HU in the pulmonary trunk, were overall scored as acceptable by one reader and good–excellent by two readers.

**Table 3.** Objective image quality—contrast opacification

Vessel location	Attenuation (HU) ± SD	Range (HU)
**Pulmonary trunk**	325 ± 72	165–531
**Right pulmonary artery**	324 ± 62	176–443
**Right lower lobe artery**	346 ± 71	192–561
**Right upper lobe artery**	357 ± 68	175–520
**Left pulmonary artery**	329 ± 61	176–451
**Left lower lobe artery**	343 ± 67	184–494
**Left lower segmental artery**	361 ± 76	206–529
**Left upper lobe artery**	353 ± 71	186–482

HU, Hounsfield unit, SD, standard deviation

The background noise, measured as the SD of the attenuation in the pulmonary trunk, was 8.8 ± 2.7 HU. The CNR in the pulmonary trunk was 32 ± 9.5, range 14–48. The CNR was similar in patients <80 kg (*n* = 35) and in patients ≥ 80 kg (*n* = 12), 33 ± 9.7 and 31 ± 8.9, respectively (*p* = 0.44).

**Figure 4.**
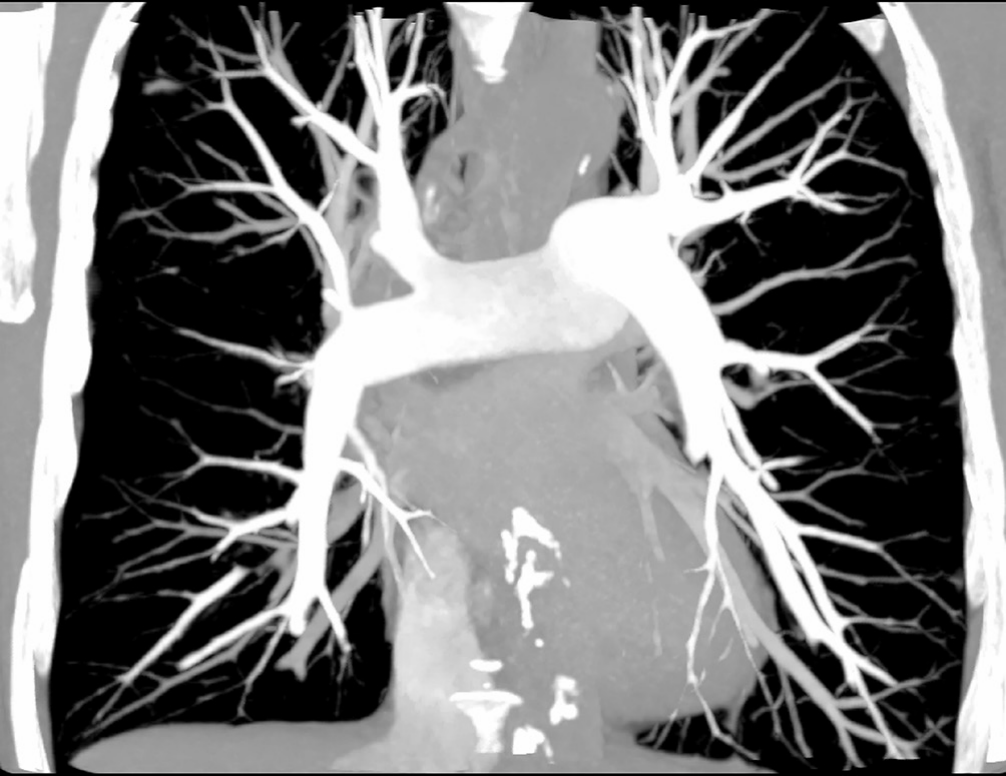
Coronal maximum intensity projection demonstrating the opacification of the pulmonary artery tree in a CTPA using 12 ml Iohexol 350 mg/ml in a 51-year-old woman. CTPA, CT pulmonaryangiography.

The visual scoring and the measured attenuation were consistent in the absence of any clear differences between central and peripheral vessels, see Tables 2 and 3. This indicates that the bolus triggered delay was sufficient for opacifying peripheral vessels. Figure 4 shows the opacification of the vascular tree including peripheral arteries in one subject.

The mean scan time was 0.8 ± 0.1 s (range 0.6–1.1 s). The delay specified in the scanner protocol was 3 s. However, measured as the difference between the acquisition time stamp in the DICOM headers (0008, 0032) of the first image in the CTPA scan and the last image of the bolus tracking, the delay was 4.3 ± 0.1 s (range 4.1–4.7 s).

The volume dose index (CTDIvol) and the dose–length product (DLP) were automatically calculated for a 32 cm body phantom for each patient. CTDIvol was 3.4 ± 0.4 mGy (range 2.1–3.7 mGy) and mean DLP was 73 ± 9.7mGy*cm (range 48–90).

## Discussion

We demonstrated that a bolus-triggered CTPA protocol using 80 kVp dual-source high-pitch acquisition provided at least adequate image quality using a mean of 17 ml CM (6 g of iodine) in 47 consecutive patients.

The high-pitch, low-kVp technique in minimizing CM dose relies on the exact timing of the scan during the CM transit in the pulmonary arteries. We combined several procedures to achieve the exact timing: preparatory saline bolus, dual flow mix of saline and CM, bolus-triggering, 80 kVp flash acquisition with lowered pitch, and free breathing.

The preparatory saline bolus, immediately before the CM bolus, was introduced from the first patient in the validation cohort, to avoid shortening of the CM bolus. We hypothesized that at the start of the injection in the antecubital fossa, a pooling could occur in collapsed veins. To avoid shortening of the CM bolus, the preparatory bolus was used to distend any collapsed vessels.

The dual-flow mix was used to achieve a sufficient flow rate for the CM bolus to compete with the unopacified blood from the inferior vena cava without changing to a lower concentration CM. The use of bolus-triggering obviated the extra CM dose associated with the test bolus.

The difference in vessel opacification between bolus-triggered CTPA and test bolus is disputed.^22,23^ With the free-breathing technique, both the delay to scan start after bolus trigger and the risk for transient interruption of CM in the pulmonary arteries due to the Valsalva maneuver are minimized.

The most important technique for reducing CM dose is lowering the kVp because it increases the attenuation of iodine.^24^ With DSCT, high-pitch mode allows an additional decrease in CM dose through the shortened bolus time. In the present study, to obtain a sufficient radiation dose, the pitch of the flash scan was lowered to 1.55.

Previous studies using high-pitch, low-kVp CTPA have used a higher CM dose than in the present study. High-pitch, 70-kVp protocol with 40 ml CM (12 grams of iodine) have been tested in some studies.^15,21^ Lu et al performed high-pitch 80 kVp CTPA with 20 ml (scan) plus 10 ml (test bolus) CM, totaling 30 ml (9 g of iodine).^14^ With conventional tube potential, high-pitch CTPA protocol have shown excellent results with 30 ml CM (10.5 g of iodine).^20^

A different approach to reducing CM dose in DSCT is dual-energy virtual monoenergetic reconstructions. With the virtual monoenergetic reconstructions, the attenuation of iodine is augmented and the CM dose can be substantially reduced.^17,18^ The most convincing results using monoenergetic reconstructions in CTPA report an iodine dose of 5.4 g on a third-generation DSCT.^17^

The average CDTI in the present study was 3.4 mGy compared to 6.8 mGy in the dual-energy study^17^ and 1.8 mGy in a previous high pitch–low kVp study.^14^ However, the radiation dose also depends on the body size of included patients.

The advantages of the high pitch–low kVp method, compared to monoenergetic reconstructions, are lower risk for respiratory artifacts, lower image noise for patients with normal body weight and low radiation dose. The disadvantages are the dependency on the exact CM timing, and the limitation of the time–current product in the flash scan mode, which may be insufficient in larger patients. The advantages of the monoenergetic approach are the lower dependency on the exact timing and the possibility to increase the radiation dose by lowering the pitch, which, however, increases the risk for respiratory artifacts. The two approaches may be complementary, and the choice made depending on patient characteristics.

The absence of a comparison group is not a significant limitation of the study. First, the goal of the study was not to prove better image quality than any other CTPA protocol, but rather to prove sufficient image quality for ruling out PE. This evaluation was performed independent of any comparison group, with the subjective image quality assessment, and supported by the objective measurements of the vascular opacification. Second, considering the large variation in CTPA protocols^9–21^ it is unclear which protocol is the appropriate one for comparison. Third, the convincing results of the image quality scores imply that the tested protocol would have scored at least as high as any comparison protocol.

Like in virtually all other CTPA protocol studies,^9–21^ a direct comparison with a reference standard could not be performed on a per patient basis; instead, the main outcome measure was the subjective image quality. The subjective image quality in CTPA mainly depends on the opacification of the pulmonary tree and artefacts affecting the image quality, with or without the presence of PE. The low number of detected PE is therefore not a significant limitation in the study.

Nevertheless, the most important limitation in the present study is that the patient cohort had to be highly selected because of the extra CM exposure associated with inclusion in the study. The patient population was not a cohort with suspected PE, which is the main application for CTPA. Suspicion of PE had to be removed from the inclusion criteria during the trial phase. A sufficient number of patients with suspicion of PE could not be included because of the difficulties in recruiting patients to a study in the acute setting, and the exclusion criteria related to risk for PC-AKI. Specifically, we could not include patients with cardiac failure. Cardiac output is known to affect the contrast timing in CT, and the pulmonary arteries may not be fully opacified in patients with substantially altered cardiac output using the present protocol. Other limitations are that the free breathing technique may not be optimal in patients with dyspnea, and that only patients with a maximum body weight of 85 kg were included. Evaluation of the CTPA protocol in a cohort referred with clinical suspicion of PE is needed and, with the present results, justifiable.

In conclusion, CTPA with good image quality using the high-pitch, low-kVp technique is possible using less than 20 ml CM.
